# Enhancing Pacific white shrimp immunity against *Vibrio parahaemolyticus* through β-glucan supplementation from marine diatoms

**DOI:** 10.14202/vetworld.2025.1047-1058

**Published:** 2025-04-30

**Authors:** Chettupon Pooljun, Pitchanee Jariyapong, Patcharapon Laksana-aut, Ikuo Hirono, Suwit Wuthisuthimethavee

**Affiliations:** 1 Akkhraratchakumari Veterinary College, Walailak University, Thasala, Nakhon Si Thammarat, 80160, Thailand; 2 Center of Excellence in Aquaculture Technology and Innovation, School of Agricultural Technology and Food Industry, Walailak University, Thasala District, Nakhon Si Thammarat, 80160, Thailand; 3 Research Center in One Health, Walailak University, Thasala District, Nakhon Si Thammarat, 80160, Thailand; 4 Department of Medical Science, School of Medicine, Walailak University, Thasala, Nakhon Si Thammarat, 80160, Thailand; 5 Graduate School of Marine Science and Technology, Tokyo University of Marine Science and Technology, Konan 4-5-7, Minato-ku, Tokyo, 108-8477, Japan

**Keywords:** immune enhancement, *lysozyme*, marine diatoms, *Penaeus vannamei*, RNA interference, transcriptomics, *Vibrio parahaemolyticus*, β-glucan

## Abstract

**Background and Aim:**

Pacific white shrimp (*Penaeus vannamei*) is a principal species in global aquaculture. However, outbreaks of *Vibrio parahaemolyticus*, the etiological agent of acute hepatopancreatic necrosis disease (AHPND), cause substantial economic losses. β-glucans derived from marine diatoms, *Chaetoceros muelleri* (CH) and *Thalassiosira weissflogii* (TH), have shown potential as immunostimulants to enhance shrimp resistance to pathogenic infections. This study aimed to evaluate the effects of β-glucans derived from CH, TH, and their combination on growth performance, immune responses, and survival of *P. vannamei* and to elucidate the underlying molecular mechanisms through transcriptomic and gene silencing approaches.

**Materials and Methods:**

Juvenile shrimp were assigned to four dietary groups for 30 days: Control (β-glucan-free), β-glucan from CH, TH, and a mixture of both (CH and TH) (CHTH). Growth performance, total hemocyte count (THC), and survival rate were evaluated. RNA-seq was performed on hepatopancreas samples after 14 days to identify differentially expressed genes (DEGs). Key immune-related DEGs were validated by quantitative reverse transcription polymerase chain reaction (qRT-PCR). Functional analysis of the *lysozyme* (*Lyz*) gene was conducted through RNA interference (RNAi), followed by *V. parahaemolyticus* challenge.

**Results:**

The CHTH diet group exhibited significantly enhanced growth metrics and the highest survival rate. Transcriptomic analysis revealed 1,902 DEGs in the CHTH group compared to control, with 915 upregulated and 987 downregulated genes. qRT-PCR validated the expression trends of selected immune-related genes, notably *Lyz*, which showed robust upregulation. RNAi-mediated *Lyz* knockdown reduced survival upon bacterial challenge, confirming its role in β-glucan-induced immunity.

**Conclusion:**

β-glucans derived from CH and TH, particularly in combination, significantly enhance growth performance and immunocompetence in *P. vannamei*. These findings underscore the potential of marine diatom-derived β-glucans as viable immunostimulants to mitigate AHPND in shrimp aquaculture, offering a sustainable alternative to antibiotic use.

## INTRODUCTION

*Penaeus vannamei*, commonly referred to as Pacific white shrimp, is a globally important aquaculture species due to its rapid growth rate, adaptability to diverse environmental conditions, and high market value. Nonetheless, the industry continues to face major obstacles, particularly from infectious diseases that can result in severe economic losses. Among the most virulent pathogens is *Vibrio parahaemolyticus*, the etiological agent of acute hepatopancreatic necrosis disease (AHPND), which has caused widespread devastation in shrimp farming across the globe in recent years [1–4]. To manage such infections, the shrimp aquaculture sector has traditionally depended on chemical treatments and antibiotics. However, the extensive use of antibiotics has raised serious concerns regarding the emergence of antibiotic resistance and potential environmental contamination. Consequently, there is a growing interest in natural alternatives, such as immunostimulants, that can enhance the shrimp’s innate immune response and thereby reduce reliance on antibiotics.

β-glucans, a class of polysaccharides present in the cell walls of fungi, bacteria, and certain algae, are well recognized for their immunostimulatory effects in various animal species [5–12]. β-glucans extracted from marine diatoms such as *Chaetoceros muelleri* (CH) and *Thalassiosira weissflogii* (TH) have shown promising potential as dietary additives for aquatic organisms, particularly shrimp [13–17]. Owing to their high β-glucan content and ease of extraction, these diatom-derived β-glucans have been incorporated into shrimp diets to promote growth, strengthen immune function, and enhance resistance to infectious diseases. A previous study in *Penaeus merguiensis* demonstrated that dietary supplementation with β-glucans from both CH and TH significantly improved growth performance and immune defense against AHPND. Histopathological examination revealed a notable increase in the number of Blasenzellen (cells containing secretory granules) and Restzellen within the hepatopancreas of shrimp administered the β-glucan-enriched diet. Following bacterial challenge, these cell types were observed at significantly higher densities compared to those in shrimp fed a control diet [18]. However, the specific molecular pathways through which β-glucans enhance hepatopancreatic immunity against AHPND remain to be fully elucidated.

Despite growing evidence that β-glucans derived from marine diatoms can enhance immune responses in shrimp, the precise immunomodulatory mechanisms remain inadequately characterized. Existing studies have largely focused on phenotypic responses such as growth performance and general immunological indices, with limited investigation into transcriptomic changes in immune-related tissues such as the hepatopancreas. Furthermore, most research has not addressed the differential efficacy of β-glucans from individual diatom species versus their combination. Critical knowledge gaps persist regarding how β-glucan structural diversity modulates gene expression patterns and contributes to pathogen resistance, particularly in the context of *V. parahaemolyticus* infections that continue to threaten shrimp aquaculture.

This study aimed to investigate the immunostimulatory effects of β-glucans extracted from CH, TH, and their combination on *P. vannamei*. Specifically, we evaluated growth performance, total hemocyte counts (THCs), and survival following dietary supplementation. To elucidate the underlying molecular mechanisms, RNA sequencing was performed on hepatopancreas tissue to identify differentially expressed immune-related genes. *Lysozym*e *(Ly*z), a key antimicrobial effector, was further assessed through quantitative reverse transcription polymerase chain reaction (qRT-PCR) and functional validation using RNA interference (RNAi) and bacterial challenge assays. This integrated approach provides mechanistic insights into how marine diatom-derived β-glucans enhance shrimp immunity and informs the development of antibiotic-free disease management strategies in aquaculture.

## MATERIALS AND METHODS

### Ethical approval

All animal experiments were approved by the Animal Ethics Committee, Walailak University (protocol no. WU-ACUC-66014).

### Study period and location

The study was conducted from February 2023 to October 2024 at the Center of Excellence for Aquaculture Technology and Innovation, School of Agricultural Technology and Food Industry, Walailak University.

### Cultivation of marine diatoms and β-glucan extraction

CH and TH were cultured in batch systems under controlled laboratory conditions following the protocol described by Pooljun *et al*. [18]. Briefly, the diatoms were grown in sterilized seawater with a salinity of 15 parts per thousand (ppt), enriched with Guillard’s f medium, at initial densities of 9.25 × 10^5^ cells/mL for CH and 2.67 × 10^5^ cells/mL for TH. Cultures were continuously aerated, maintained at 25 ± 1°C, and exposed to fluorescent light at 5,000 lux. After 5 days, CH and TH reached peak densities of 1.32 × 10^7^ cells/mL and 1.24 × 10^6^ cells/mL, respectively. Diatoms were precipitated using polyaluminium chloride and centrifuged at 3,700× *g* for 15 min using the Multifuge X Pro Centrifuge Series (Thermo Fisher Scientific, USA). Cell pellets were washed with distilled water and centrifuged again at 3,700× *g* for 15 min. β-glucans were extracted using a modified hot-water method, as previously described by Chiovitti *et al*. [19].

### Experimental diets

The control diet consisted of a commercial shrimp feed (PHOCA 803P, Phoca Feed Co., Ltd.) containing 36% crude protein. Experimental diets were prepared by incorporating β-glucan from CH, TH, or a combination of both (CH and TH) (CHTH) into the commercial feed at an inclusion level of 0.2%, which has been established as optimal for shrimp in prior studies by Pooljun *et al*. [18], Mameloco and Traifalgar [20], Bai *et al*. [21], Chang *et al*. [22], and Ochoa-Álvarez *et al*. [23]. The ingredients were mixed with minimal water, and the resulting pellets were air-dried at room temperature (25°C) until moisture content dropped below 10%. Pellets were subsequently sprayed with 1.5% fish oil to prevent nutrient leaching [24] and stored at −20°C until use.

### Experimental animals

Pacific white shrimp (*P. vannamei*) with an average weight of 5.10 ± 0.16 g were obtained from a commercial farm in Nakhon Si Thammarat province, Thailand. Shrimp were transferred to aerated seawater tanks and acclimated for 1 week at room temperature. During acclimation, they were fed a commercial diet at 5% of body weight 3 times daily.

### Experimental design and management

Following acclimation, shrimp with uniform body weights were randomly assigned to four groups (n = 80 per group). Each group was subdivided into eight tanks containing 10 shrimp in 100-L aerated tanks. Environmental parameters – including temperature (28 ± 1°C), salinity (25 ppt), pH (7.8–8.2), total ammonia nitrogen (<1.0 mg/L), and nitrite (<0.5 mg/L) – were closely monitored and maintained. Water was partially exchanged (20%) daily.

The groups were assigned diets as follows: Group 1 received the β-glucan-free control diet (CTRL), Group 2 received β-glucan from CH, Group 3 from TH, and Group 4 received the combined β-glucan diet (CHTH). Diets were administered for 30 days. In each group, six tanks (n = 60) were used for immunological assays and two tanks (n = 20) for growth performance assessment.

Hemolymph and hepatopancreas samples were collected from 10 shrimp per group at 1, 3, 7, 14, and 30 days post-feeding for THC and immune-related gene expression analysis. On day 14, hepatopancreas samples were selected for RNA sequencing under blinded conditions. Shrimp were weighed individually on days 0 and 30 to evaluate growth parameters, including final weight, weight gain, average daily growth (ADG), and survival. All experiments were conducted in a double-blind design to minimize bias.

### RNA sequencing and bioinformatic analysis

Total RNA was extracted from the hepatopan-creas of 10 shrimp per group after 14 days of feeding using GENEzol reagent (Geneaid, Taiwan), following the manufacturer’s instructions. RNA quality was assessed using a NanoDrop 2000c spectrophotometer (Thermo Fisher Scientific, USA), an Agilent 2100 Bioanalyzer, and 1% agarose gel electrophoresis. Samples with RNA integrity number > 7 were pooled and submitted to Novogene Co., Ltd. (Beijing, China) for sequencing on the Illumina NovaSeq platform (Illumina, Inc. San Diego, California, USA).

Raw reads were processed using custom Perl scripts to remove adapter sequences, poly-N regions, and low-quality reads. Clean reads were aligned to the *P. vannamei* reference genome (GCF_003789085.1_asm378908v1) using HISAT2 (v2.0.5) (http://ccb.jhu.edu/software/hisat2) and assembled with StringTie (v1.2.3b) (http://ccb.jhu.edu/software/stringtie). Gene expression levels were calculated as Fragments Per Kilobase of transcript per million mapped reads [25]. Differentially expressed genes (DEGs) were identified using the edgeR package (v3.22.5) [26], with thresholds set at |log_2_ (fold change)| ≥ 1 and adjusted p ≤ 0.05.

Functional enrichment analysis of DEGs was conducted using the clusterProfiler R package (http://bioconductor.org/packages/clusterProfiler). Gene Ontology terms and Kyoto Encyclopedia of Genes and Genomes (KEGG) pathways with adjusted p < 0.05 were considered significantly enriched. KEGG data were sourced from the official KEGG database (http://www.genome.jp/kegg/).

### Gene expression analysis

Expression levels of selected immune-related DEGs identified by RNA-seq were validated through qRT-PCR. Genes of interest – c-type lectin, chitotriosidase, Lyz, phenoloxidase-activating factor, and serine protease – were selected based on their upregulation in shrimp fed β-glucan-supplemented diets. One microgram of total RNA was reverse-transcribed into complementary DNA using iScript™ Reverse Transcription Supermix (Bio-Rad, USA). qRT-PCR was performed using a CFX96 system (Bio-Rad) and 5× HOTFIREPol^®^ EvaGreen^®^ qPCR Mix Plus (Solis Biodyne, Estonia). Beta-actin served as the reference gene. Each assay included negative controls and was conducted in triplicate. Melt curve analysis was used to confirm amplification specificity. Fold changes were calculated using the 2^−ΔΔCT method [27], and statistical significance was assessed using Student’s t-test. Primer sequences are listed in Table 1.

**Table 1:** Sequences of primers used for immune-related gene amplification.

Name	GenBank accession number	Primer sequence (5’–3’)	E (%)	r^2^	Slope	Tm (°C)	Purpose
C-type lectin	XM_070127396.1	(F) 5’- ATTCTCGCTGGGATGTCTGC-3’ (R) 5’- GCGTGACTTCTTGGCTCTCT-3’	94.2	0.981	−3.425	87	qRT-PCR
Chitotriosidase Chitinase-3	XM_070122235.1	(F) 5’- GATGGTGTGCTACTTCGGCT-3’ (R) 5’- TGGCATTCTGCTGCTTGAGA-3’	96.2	0.998	−3.416	87.2	qRT-PCR
Lysozyme	XM_027357011.2	(F) 5’- TAATCAGCAAGGAAGGGCCG-3’ (R) 5’- ACTTCATCTGGCATGACGCA-3’	108.3	0.992	−3.139	87.4	qRT-PCR
Phenoloxidase- activating factor	XM_027364048.2	(F) 5’- GAGACGACTGGAACGAGCAA-3’ (R) 5’- GTGGTCTCCCAGCGAAATGA-3’	104.0	0.993	−3.229	87.1	qRT-PCR
Serine protease	XM_070131467.1	(F) 5’- ATGCCTTGACAGACTTCGCA-3’ (R) 5’- CGCTGCTCTGATAGGTCCAG-3’	96.3	0.994	−3.413	91	qRT-PCR
β-actin	AF200705.2	(F) 5’- CCACGAGACCACCTACAAC -3’ (R) 5’- AGCGAGGGCAGTGATTTC -3’	98.9	0.998	−3.349	88.4	qRT-PCR
Lysozyme-T7		(F) 5’- TAATACGACTCACTATAGGGTAA TCAGCAAGGAAGGGCCG-3’ (F) 5’- TAATACGACTCACTATAGGGA CTTCATCTGGCATGACGCA-3’					RNAi
*GFP*-T7		(F) 5’- TAATACGACTCACTATAGGGATG GTGAGCAAGGGCGAGGA-3’ R) 5’- TAATACGACTCACTATAGGGTT ACTTGTACAGCTCGTCCA-3’					RNAi

E=qPCR efficiency, r^2^=Correlation coefficient of standard curves, Tm=Melting temperature, qRT-PCR=Quantitative reverse transcription polymerase chain reaction, *GFP=Green fluorescent protein*

### Production of double-stranded RNA (dsRNA)

The *Lyz* gene, which showed high expression post β-glucan supplementation, was selected for RNAi. T7 promoter-linked primers for *Lyz* and *Green fluorescent protein* (GFP) genes [28] were used with the T7 RiboMAX™ Express RNA Production System (Promega, USA) for dsRNA synthesis. Products were purified through phenol-chloroform extraction and quantified using a NanoDrop 2000c spectrophotometer. dsRNA was stored at −20°C until use.

Thirty shrimp were randomly assigned to three groups (n = 10) and injected with 1 µg/g body weight of either *Lyz* dsRNA, GFP dsRNA, or phosphate-buffered saline (PBS, control). At 24 and 48 h post-injection, RNA was extracted from hemocytes, gills, and hepatopancreas to assess *Lyz* gene silencing through qRT-PCR.

### Bacterial challenge

The *V. parahaemolyticus* AHPND strain (AHNND) was cultured as previously described by Pooljun *et al*. [29] in tryptic soy broth with 1.5% NaCl at 32°C for 16–18 h. Cultures were centrifuged, washed with sterile saline (0.85% NaCl), and resuspended. Optical density (OD_600_ = 1.00) was used to estimate a concentration of ~10^8^ colony-forming units (CFU)/mL, followed by serial dilution to obtain concentrations of 10^8^–10^3^ CFU/mL. The LD_50_ was determined by immersion challenge in 15 naïve shrimp per dilution. A final concentration of 10^6^ CFU/mL was used for the experimental challenge.

Eighty shrimp were assigned to two diet groups (CHTH or control), with 40 shrimp each. Within each diet group, shrimp were injected with either *Lyz* or GFP dsRNA (1 µg/g). At 48 h post-injection, shrimp were immersed for 30 min in water containing the LD_50_ dose of *V. parahaemolyticus* and then transferred to clean tanks. Survival was monitored daily for 10 days. Survival differences were analyzed using the Mantel-Cox (log-rank χ²) test through GraphPad Prism (GraphPad Software, San Diego, CA, USA). Hepatopancreas samples from deceased shrimp were tested through polymerase chain reaction (PCR) for *V. parahaemolyticus* detection, following Tinwongger *et al*. [30].

### Statistical analyses

All data were analyzed using the Statistical Package for the Social Sciences version 22.0 (IBM Corp., Armonk, NY, USA). Results are presented as mean ± standard deviation. One-way analysis of variance (ANOVA) followed by Tukey’s multiple comparison test was used to assess differences among groups at a significance level of p < 0.05. Two-way ANOVA was used to evaluate interactions between treatment and time on immune parameters. Survival data were analyzed using Kaplan–Meier survival curves and log-rank (Mantel-Cox χ²) tests through GraphPad Prism.

## RESULTS

### Shrimp growth performance and THC

At day 30 following oral administration, shrimp fed diets supplemented with β-glucans derived from marine diatoms (CH, TH, and CHTH) exhibited significantly higher final weights (9.85 ± 0.64 g, 9.82 ± 0.57 g, and 9.87 ± 0.51 g, respectively), weight gains (4.72 ± 0.12 g, 4.76 ± 0.15 g, and 4.78 ± 0.12 g, respectively), and average daily growth rates (0.157, 0.159, and 0.159, respectively) compared to shrimp fed the control diet. However, no significant differences were observed among the β-glucan-supplemented groups (CH, TH, and CHTH) (Table 2).

**Table 2:** Final mean (± SD) values for final weight, weight gain, average daily growth, and survival rate of *P. vannamei* fed a commercial control diet or diets supplemented with 2 g/kg of β-glucan derived from CH, TH, and CHTH.

Parameter	Treatment			

Control	CH	TH	CHTH
Initial weight (g)	5.07 ± 0.17^a^	5.09 ± 0.18^a^	5.09 ± 0.14^a^	5.11 ± 0.16^a^
Final weight (g)	8.80 ± 0.75^a^	9.82 ± 0.57^b^	9.85 ± 0.64^b^	9.87 ± 0.51^b^
Weight gain (g)	3.72 ± 0.17^a^	4.72 ± 0.12^b^	4.76 ± 0.15^b^	4.78 ± 0.12^b^
ADG	0.124^a^	0.157^b^	0.159^b^	0.159^b^
Survival rate (%)	85^a^	90^a^	90^a^	95^a^

^a,b^Values with different superscripts within the same row differ significantly (p < 0.05). SD=Standard deviation, ADG=Average daily growth, *P. vannamei*=*Penaeus vannamei*, CH=*Chaetoceros muelleri*, TH=*Thalassiosira weissflogii*, CHTH=*Chaetoceros muelleri* and *Thalassiosira weissflogii*

The final survival rates were 85% for the control group and 90%, 90%, and 95% for the CH, TH, and CHTH groups, respectively. Statistical analysis revealed no significant differences in survival among the groups (p = 0.78, Chi-square test). Interestingly, shrimp fed with β-glucan derived from TH exhibited a 5% higher survival rate than those fed with CH or the mixed β-glucan diet and 10% higher than the control group (Table 2).

**Figure 1:**
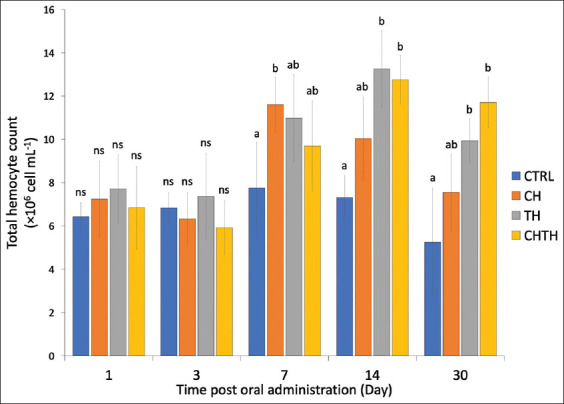
Total hemocyte count of shrimp fed diets containing β-glucan derived from *Chaetoceros muelleri*, *Thalassiosira weissflogii*, and a mixture of both diatoms compared to a control diet at different time points. Each bar represents the mean ± standard deviation (n =10). Bar labeled with different letters indicate statistically significant differences based on Tukey’s multiple comparison test (p < 0.05), while “ns” denotes no significant difference.

Following 1 day of feeding, the THC increased slightly in all experimental groups compared to the control group, ranging from 6.84 ± 1.91 × 10^6^ to 7.24 × 10^6^ cells/mL, though differences were not stati-stically significant. By day 3, THC values decreased slig-htly (5.92 ± 1.74 × 10^6^ to 6.83 ± 0.71 × 10^6^ cells/mL), again with no significant group differences. However, at day 7, shrimp fed β-glucan from CH exhibited a significantly higher THC compared to the control group (13.61 ± 2.25 × 10^6^ vs. 7.76 ± 2.10 × 10^6^ cells/mL). Although higher THC levels were also observed in the TH and CHTH groups (9.98 ± 2.42 × 10^6^ and 11.69 ± 2.59 × 10^6^ cells/mL, respectively), these differences were not statistically significant. On days 14 and 30, shrimp in all β-glucan-supplemented groups consistently exhibited elevated THC levels compared to the control (Figure 1).

### Transcriptome analysis

Following RNA sequencing and quality filtering, clean read counts of 12.76 M (CTRL), 11.88 M (CH), 11.69 M (TH), and 11.29 M (CHTH) were obtained at day 14 (Table 3). *De novo* assembly yielded an average transcriptome length of 79,367,040 bp across 13,206 unigenes. All sequencing data were submitted to the Sequence Read Archive (SRA; NCBI) under accession numbers SRX27390263, SRX27390264, SRX27390265, and SRX27390266, respectively (SRA-PRJNA1212290).

**Table 3:** Summary statistics of the hepatopancreas transcriptome of *P. vannamei* after feeding with β-glucan derived from different marine diatoms.

Sample	Raw reads	Raw bases	Clean reads	Clean bases	Q20	% GC
Control	86233820	12.94G	85091372	12.76G	98.22	48.90
CH	80418622	12.06G	79178132	11.88G	98.13	48.48
TH	79330916	11.90G	77964028	11.69G	97.60	49.03
CHTH	76605650	11.49G	75234626	11.29G	98.03	49.00
Average	80647252	12.10G	79367040	11.91G	98.00	48.85

*P. vannamei*=*Penaeus vannamei*, CH=*Chaetoceros muelleri*, TH=*Thalassiosira weissflogii*, CHTH=*Chaetoceros muelleri* and *Thalassiosira weissflogii*

### Differential gene expression in the hepatopancreas

**Figure 2:**
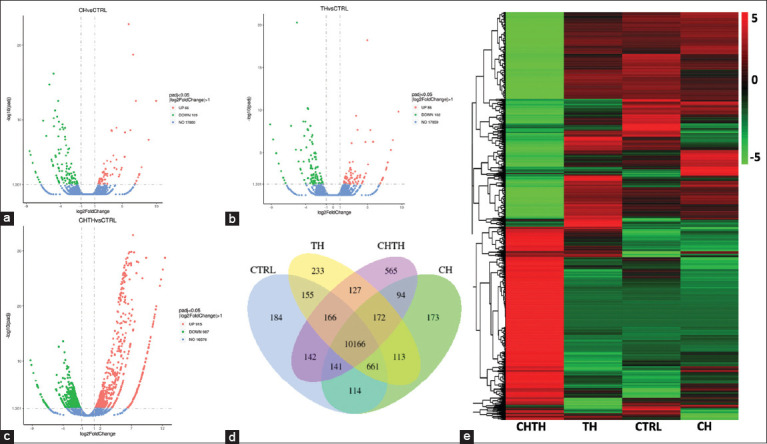
Expression profile revealed by short-read RNA sequencing data. (a–c) Display volcano plot of differentially expressed transcripts between control diet and diets supplemented with different sources of β-glucan (CH, TH, and CHTH, respectively) in *L. vannamei* hepatopancreas at 14 days after administration. The x-axis indicates the fold change in gene expression between samples, whereas the y-axis indicates the statistical significance of the differences. Red dots represent upregulated genes and green dots represent downregulated genes; (d) Venn diagram illustrates the number of common and unique differentially expressed genes among the experimental groups (CH, TH, and CHTH) compared with the control group (CTRL); (e) Heatmap of transcriptome sequence data across all groups. *CH=Chaetoceros muelleri*, TH*=Thalassiosira weissflogii*, CHTH*=Chaetoceros muelleri* and *Thalassiosira weissflogii*.

DEGs presumed to be β-glucan-responsive in the shrimp hepatopancreas were normalized and compared to control samples using the edgeR program with thresholds set at absolute log_2_-fold change and false discovery rate (FDR) < 0.001. A total of 12,206 unigenes were differentially expressed across the β-glucan-fed groups. Specifically, 175 DEGs were identified between the CTRL and CH groups (66 upregulated, 109 downregulated) (Figure 2a) and 188 DEGs between the CTRL and TH groups (86 upregulated, 102 downregulated) (Figure 2b). The CHTH group presented 1,902 DEGs (915 upregulated, 987 downregulated) relative to the control (Figure 2c).

Gene overlap analysis across libraries revealed that 11,729 (CTRL), 11,634 (CH), 11,793 (TH), and 11,573 (CHTH) genes were identified, with 10,166 genes (76.98%) shared across all groups. A total of 1,155 genes (8.75%) were uniquely expressed in individual treatment groups: 184 (1.39%) in CTRL, 173 (1.31%) in CH, 233 (1.76%) in TH, and 565 (4.28%) in CHTH (Figure 2d). The proportion of genes uniquely expressed in CH, TH, and CHTH libraries compared to all detected genes was 971/13,206 (7.35%), indicating considerable expression modulation by β-glucan supplementation. The DEG expression patterns were most notably altered in the CHTH group compared to CTRL, CH, and TH (Figure 2e).

### Validation of RNA sequencing by qRT-PCR

**Figure 3:**
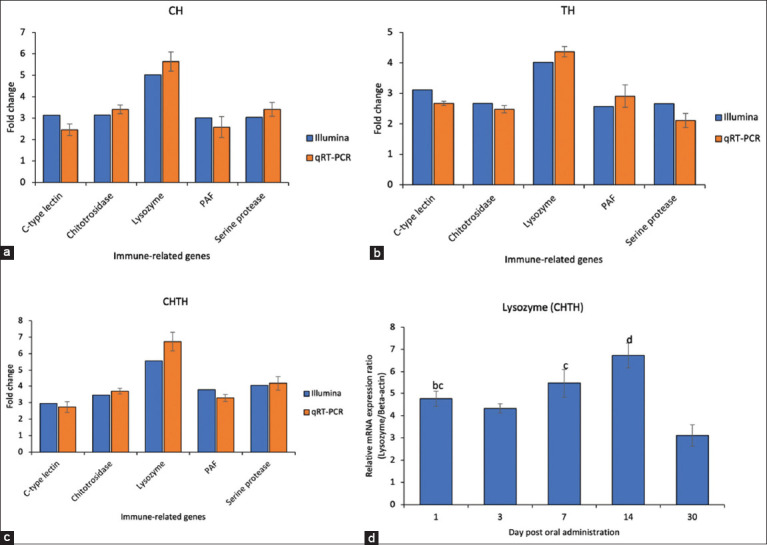
Comparison of differential expression of five immune-related genes determined by Illumina sequencing and qRT–PCR in the β-glucan supplemented diet groups. The relative transcript levels (fold changes) of CH (a), TH (b), and CHTH (c) were determined by qRT-PCR using beta-actin as the internal control. In addition, the effects of the mixed β-glucan supplemented diet (CHTH) on the relative mRNA expression levels of the *Lysozyme* gene at different time points (at days 1, 3, 7, 14, and 30 post-administration) are shown (d). Each bar represents the mean ± standard deviation (n = 10), and bars with different letters indicate significant differences according to Tukey’s multiple comparison test (p < 0.05). qRT–PCR=Quantitative reverse transcription polymerase chain reaction, *CH=Chaetoceros mueller*i, *TH=Thalassiosira weissflogi*, CHTH*=Chaetoceros muelleri* and *Thalassiosira weissflogii*.

To validate RNA-seq results, five immune-related DEGs – c-type lectin, chitotriosidase, Lyz, phenoloxidase-activating factor (PAF), and serine protease – were assessed using qRT-PCR. The expression profiles obtained by qRT-PCR were consistent with the RNA-seq data (Figure 3a-c), confirming the reliability of transcriptome results. Among these genes, *Lyz* showed significant upregulation in shrimp fed the β-glucan-supplemented diet compared to the control.

Temporal analysis of *Lyz* mRNA expression in shrimp hepatopancreas (CHTH group) showed dynamic regulation: expression was approximately 4.5-fold higher than control on day 1, slightly decreased to 4.2-fold by day 3, increased again on day 7, and peaked at ~7-fold on day 14. By day 30, expression had markedly declined (Figure 3d).

### *Lyz* knockdown reduces survival following bacterial challenge

**Figure 4:**
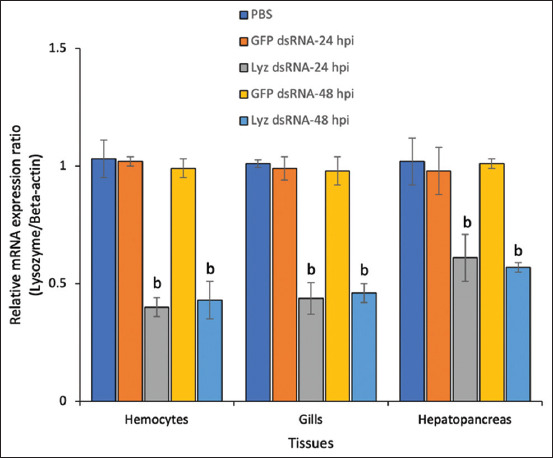
RNA interference assay of *lysozyme* (*Lyz*). Relative expression levels of the *Lyz* gene following *Lyz* dsRNA and *GFP* dsRNA delivery. Each bar represents the mean ± SD (n = 5) of the qRT–PCR analysis. Bars labeled with different letters indicate statistically significant differences according to Tukey’s multiple comparison test (p < 0.05). qRT–PCR=Quantitative reverse transcription polymerase chain reaction, dsRNA=double-stranded RNA, *GFP*=*Green fluorescent protein*.

To confirm the functional role of *Lyz* in β-glucan-mediated immunity, RNAi was employed. As shown in Figure 4, *Lyz* gene knockdown was effectively achieved in hemocytes, gills, and hepatopancreas, with expression reduced by ~60% and ~57% at 24 and 48 h post-injection, respectively. In gills and hepatopancreas, expression was suppressed by over 50% at 24 h and fell below 40% at 48 h.

Following RNAi and subsequent challenge with *V. parahaemolyticus*, shrimp in both β-glucan-fed and control diet groups exhibited >50% mortality within 5 days. By day 10, β-glucan-fed shrimp had a higher survival rate than the control group, although the difference was not statistically significant (χ^2^ = 0.0176, p = 0.8943).

**Figure 5:**
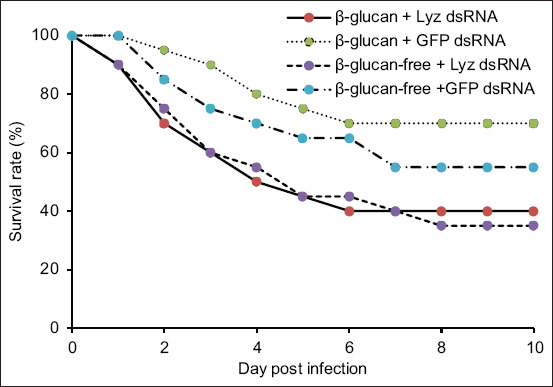
Cox Proportional Hazards and Kaplan–Meier survival analysis of *Penaeus vannamei* fed diets with or without β-glucan supplementation and treated with *lysozyme* dsRNA or *GFP* dsRNA following *Vibrio parahaemolyticus* infection. Differences in survival rates among groups were analyzed using Kaplan–Meier log-rank χ^2^ tests (*p < 0.05). dsRNA=double-stranded RNA, *GFP*=*Green fluorescent protein*. β-glucan + *Lyz* dsRNA/β-glucan + *GFP* ds RNA (HR = 2.655 (1.004–6.752), p = 0.034). β-glucan + *Lyz* dsRNA/β-glucan-free + *Lyz* ds RNA (HR = 0.951 (0.434–2.083), p = 0.894). β-glucan + *Lyz* dsRNA/β-glucan-free + *GFP* ds RNA (HR = 1.627 (0.688–3.842), p = 0.240). β-glucan + *GFP* dsRNA/β-glucan-free + *Lyz* ds RNA (HR = 0.350 (0.141–0.867), p = 0.021). β-glucan + *GFP* dsRNA/β-glucan-free + *GFP* ds RNA (HR = 0.600 (0.218–1.654), p = 0.311).

**Figure 6:**
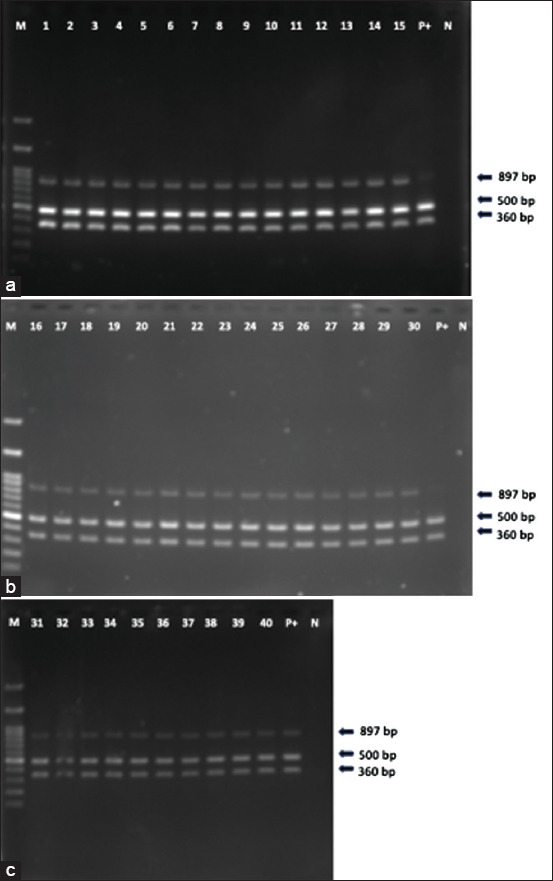
Agarose gel electrophoresis of hepatopancreas specimens from dead shrimp infected with *Vibrio parahaemolyticus* acute hepatopancreatic necrosis disease (AHPND) strain. Panels A (a), B (b), and C (c) show three specific bands at 897, 500, and 360 bp. Lane M: 100-bp DNA ladder; Lane P+: Positive control (*V. parahaemolyticus* AHPND strain DNA from pure culture); Lane N: Negative control (distilled water); Lanes 1–13: Samples from β-glucan-free diet + dsRNA group; Lanes 14–22: Samples from β-glucan-free diet + ds*GFP* group; Lanes 23–34: Samples from the β-glucan + *Lyz*-sdRNA group; Lanes 35-40: Samples from the β-glucan + *Lyz*-sd*GFP* group. *Lyz*=*Lysozyme*, dsRNA=double-stranded RNA, *GFP*=*Green fluorescent protein*.

In shrimp not subjected to *Lyz* knockdown, β-glucan supplementation significantly improved survival following infection (χ^2^ = 4.482, p = 0.0343). Although shrimp fed the β-glucan diet showed a higher survival rate (70%) than those on the control diet (55%), the difference was not statistically significant (χ^2^ = 1.025, p = 0.3113) (Figure 5). Notably, enhanced *Lyz* expression correlated with increased survival. PCR analysis confirmed that mortality was attributable to *V. parahaemolyticus* infection in all deceased specimens (Figure 6).

## DISCUSSION

Dietary supplementation with β-glucans derived from marine diatoms significantly enhanced shrimp growth performance, comparable to the effects of β-glucans sourced from bacterial, yeast, and fungal cell walls [31, 32]. Improvements were observed in average daily growth (ADG), weight gain, and final weight in comparison to the control group. However, survival rates did not differ significantly among the control and β-glucan-fed groups. These findings confirm that β-glucans from marine diatoms are both safe and effective for promoting the growth of Pacific white shrimp, consistent with outcomes previously reported in banana shrimp and black tiger shrimp [18, 21, 33, 34]. Survival rates ranged from 85% in the control group to 95% in the CHTH group. Although these differences were not statistically significant, the observed trends – particularly in the CHTH group – suggest species-specific variations in the bioactivity of β-glucans derived from CH and TH. Earlier studies have indicated that β-glucans extracted from different diatom species may differ in molecular structure and immunomodulatory capacity [18, 35].

Furthermore, β-glucans are recognized for their immunostimulatory potential and ability to modulate gut microbiota, thereby improving nutrient absorption and contributing to enhanced growth and survival [18, 31, 34, 36–38]. The significant elevation in THC observed in shrimp fed β-glucan-supplemented diets underscores the role of marine diatom-derived β-glucans as potent immune enhancers. Daily administration of β-glucans, whether from a single species or in combination, resulted in significantly higher THC compared to unsupplemented diets [39, 40]. These results are in line with a previous study by Mameloco and Traifalgar [20], which demonstrated that shrimp receiving immunostimulants daily exhibited significantly elevated THC compared to those fed at intervals of 3 or 7 days.

Transcriptome analysis remains a powerful approach to elucidate gene expression dynamics and molecular signaling pathways in vertebrates and invertebrates. In shrimp, the hepatopancreas is essential for digestion and metabolism and for orchestrating innate immune responses [37, 41–44]. In this study, comparative transcriptomic profiling conducted after a 14-day feeding trial with β-glucan-supplemented diets revealed substantial differential gene expression, particularly in shrimp fed the combined β-glucan diet (CHTH). This group exhibited 915 upregulated and 987 downregulated genes compared to the control group. In contrast, shrimp fed individual β-glucans derived from CH or TH demonstrated fewer DEGs, indicating a more moderate transcriptional response.

Interestingly, the structural synergy of the mixed β-glucans comprising both the branched β-1,3 and β-1,6-linked glucose polymers from CH and the linear β-1,3-glucan polymer from TH [35] may account for the stronger immunostimulatory effect observed. Among the significantly upregulated immune-related genes were *C-type lectin*, *Lyz*, and *serine protease*, all of which are critical components of the shrimp’s innate immune system.

*Lyz*, a well-characterized antimicrobial enzyme found in animals, plants, and microorganisms [45], is a central component of the innate immune defense against bacteria, fungi, and viruses [46, 47]. In shrimp, *Lyz* is primarily produced by hemocytes in response to pathogenic threats, and its expression has also been detected in the heart, lymphoid organ, gills, muscle, and hepatopancreas [48]. Our findings demonstrated a marked upregulation of *Lyz* mRNA in the hepatopancreas as early as day 3 post β-glucan administration (Figure 3d), consistent with earlier studies showing increased transcript levels of *Lyz* within 24 h to 60 days following β-glucan exposure [39, 49]. These results suggest that *Lyz* expression is highly responsive to β-glucan stimulation and plays an essential role in host defense.

RNAi was utilized to evaluate the functional significance of *Lyz* in β-glucan-mediated immunity. *Lyz* transcript levels were significantly reduced in hemocytes, gills, and hepatopancreas within 24 h post-dsRNA injection and remained suppressed at 48 h (Figure 4). These results confirm the effective knockdown of *Lyz* gene expression by RNAi, consistent with prior findings reporting dsRNA-mediated suppression of gene expression lasting 24–72 h [50, 51].

The reduction in *Lyz* expression correlated with lower survival rates following *V. parahaemolyticus* infection, indicating its pivotal role in immune defense. Notably, shrimp fed a β-glucan-supplemented diet for 14 days exhibited higher survival rates even after *Lyz* knockdown, compared to those on a control diet. This suggests that while *Lyz* is a major effector, other immune pathways stimulated by β-glucans may contribute to protection against bacterial challenge.

## CONCLUSION

This study demonstrated that dietary supplementation with β-glucans derived from the marine diatoms CH and TH significantly enhanced the growth performance, immunological parameters, and transcriptional responses of *P. vannamei*. Shrimp fed β-glucan-supplemented diets, particularly the combination of both diatoms (CHTH), exhibited superior weight gain, increased THCs, and robust differential expression of immune-related genes. Transcriptome analysis revealed a pronounced upregulation of key immune effectors, including *Lyz*, *c-type lectin*, and *serine protease*, in the CHTH group. Functional validation through RNAi further confirmed the pivotal role of *Lyz* in β-glucan-mediated immune protection against *V. parahaemolyticus*, as gene silencing significantly reduced shrimp survival following bacterial challenge.

The primary strength of this study lies in its integrative approach, combining growth performance assessment, immunoassays, high-throughput transcriptomics, and functional gene silencing to evaluate the immunostimulatory efficacy of marine diatom-derived β-glucans comprehensively. Furthermore, the inclusion of both single and combined β-glucan sources provides novel insights into the synergistic potential of structurally diverse β-glucan molecules.

However, this study is not without limitations. The duration of dietary administration was limited to 30 days, which may not fully capture long-term immunomodulatory effects or microbiota interactions. In addition, the study focused on hepatopancreatic gene expression, while overlooking other critical immune tissues such as the lymphoid organ or hemocytes. The potential role of β-glucans in modulating intestinal microbiota and metabolic pathways was also not addressed and warrants further exploration.

Future research should aim to elucidate the receptor-mediated signaling pathways involved in β-glucan recognition and downstream immune activation. Longitudinal studies evaluating the effects of prolonged supplementation, dose optimization, and interactions with gut microbiota could provide deeper insights into the holistic impact of these immunostimulants. Moreover, evaluating β-glucan efficacy under commercial farming conditions would facilitate the practical application of these findings in shrimp aquaculture.

In conclusion, marine diatom-derived β-glucans represent a promising, eco-friendly alternative to antibiotics in shrimp farming, offering both immunological and growth-enhancing benefits. Their strategic inclusion in aquafeeds could substantially contribute to sustainable and disease-resilient shrimp production systems.

## AUTHORS’ CONTRIBUTIONS

CP, PJ, and SW: Conceptualized and designed the study. CP, PJ, and PL: Methodology. CP, PJ, and IH: Data curation and interpretation. CP, PJ, SW: Drafted and revised the manuscript. All authors have read and agreed to the final version of the manuscript.
